# Supplementary material for: Re-evaluating the functional landscape of the cardiovascular system during development
Source: Biol Open. 2017 Oct 5;6(11):1756–70. doi: 10.1242/bio.030254 (PMC5703621; doi:10.1242/bio.030254)
Supplement: Supplementary information [file biolopen-6-030254-s1.pdf]

Figure S1

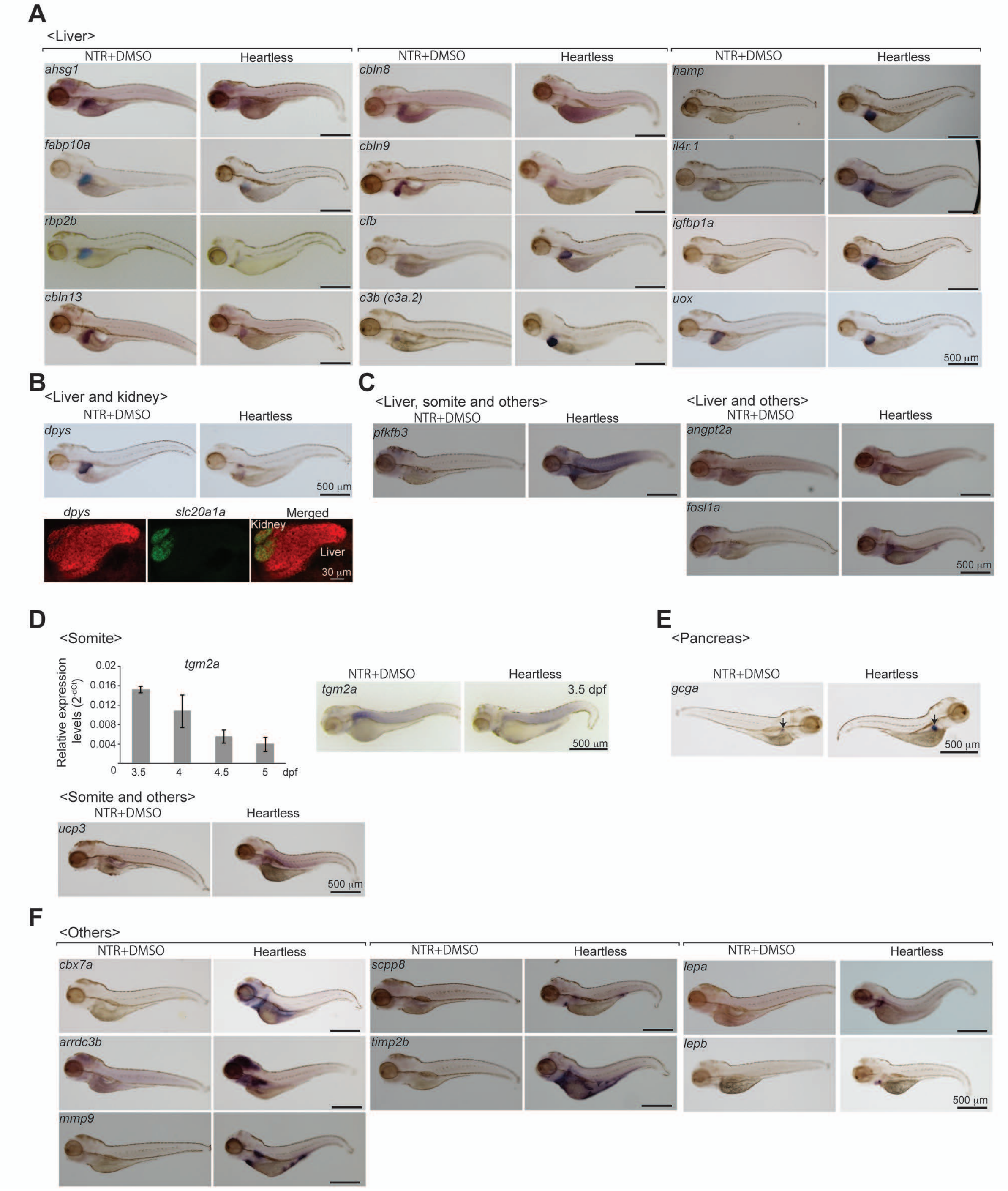

Figure S1. WISH expression patterns of the genes differentially expressed in "heartless". The patterns are shown for *Tg*(*cmlc2:mcherry-NTR*) treated by DMSO (NTR+DMSO) and "heartless" larvae at 4.5 dpf for each gene. **A.** Liver genes. **B.** Liver/kidney genes. The renal expression of dpys in 4.5 dpf larva is shown by co-staining with a marker for proximal convoluted tubule, slc20a1a. **C.** Genes expressed in the liver/somite/others (pfkfb3) and the liver/others (angpt2a and fosl1a). **D.** Somite gene. qRT-PCR result (top graph) shows the higher expression of tgm2a at 3.5 dpf. **E.** Pancreas gene. **F.** Genes expressed in undefined tissues/organs (Others). Scale bars, 500 µm.

Figure S2

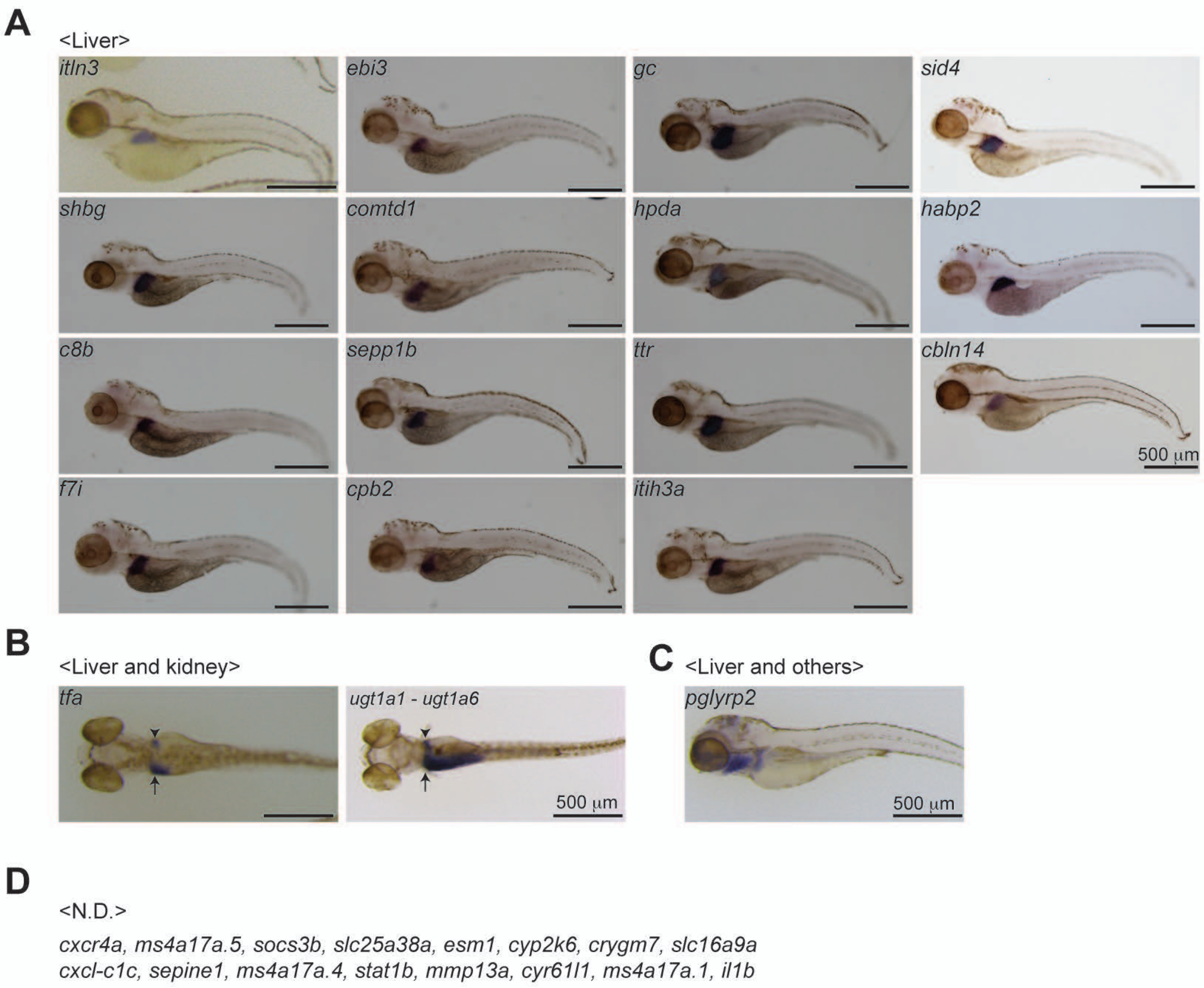

Figure S2. WISH expression patterns of the other genes. A. Liver genes. **B.** Genes expressed in both the liver and kidney. **C.** Genes expressed in the liver and tissues/organs that are undefined (Others). **D.** Genes whose expressions are not determined (N.D.). Scale bars, 500 µm.

Biology Open • Supplementary information

Biology Open (2017): doi:10.1242/bio.030254: Supplementary information

Figure S3

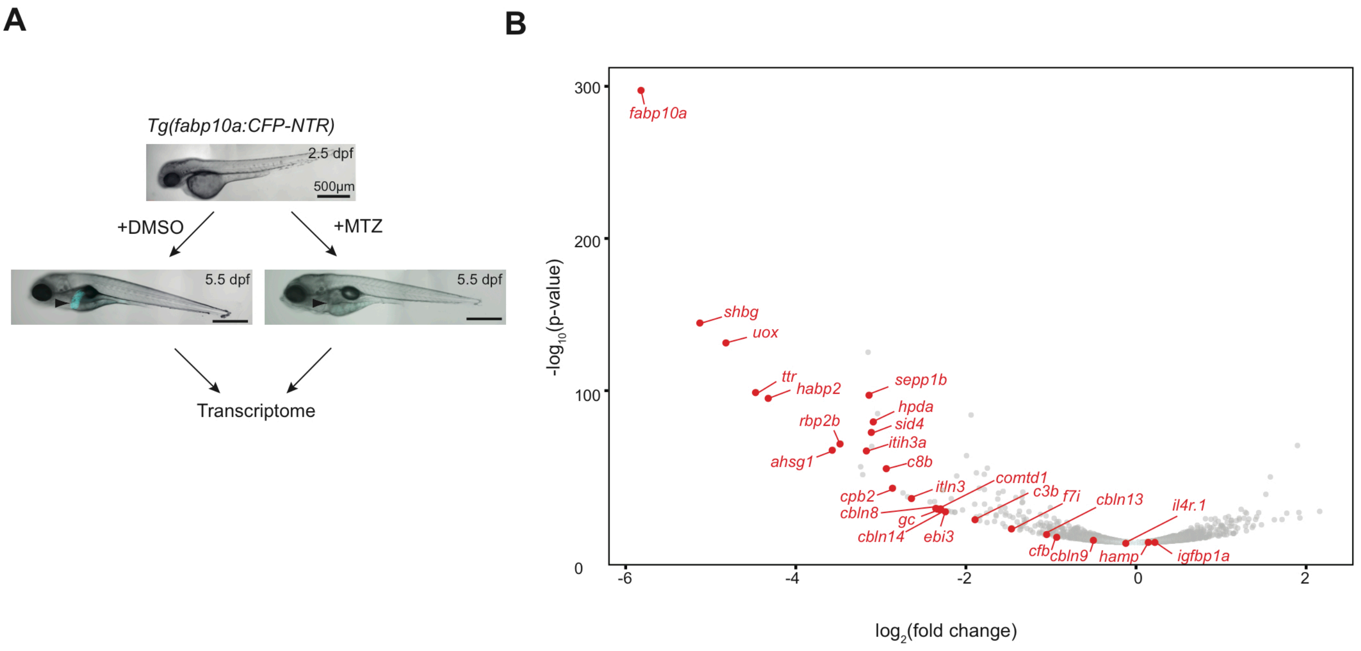

Figure S3. Liver genes. A. Generation of “liverless”. Scale bars, 500 µm. Arrowheads denote fluorescent (Cyan) liver. **B.** Volcano plot of the body-wide transcriptome data of “liverless” showing the liver genes (red). Their expression was significantly reduced in “liverless”, except two genes (igfbp1a and hamp). However, their expression in the liver was confirmed by WISH (Fig. S1A). It is possible that they are upregulated in the small residual liver tissues in “liverless”.

Biology Open • Supplementary information

Figure S4

A

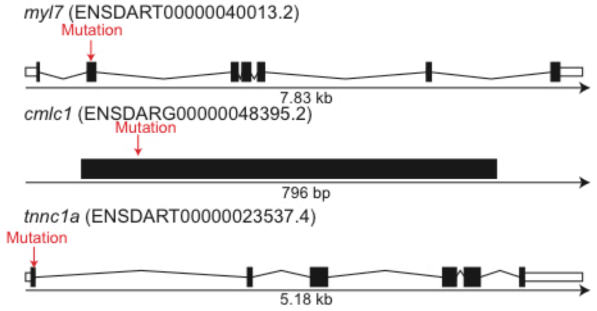

B

```
                                     gRNA Target
myl7              GCCAAGAGGGGGAAAACTGCTCAAAGAGGCTC
myl7(+5bp)        GCCAAGAGGGGAAAAACTGCTCggcacAAAGA (+5bp)
myl7(+7bp)        GCCAAGAGGGGGAAAACTGCTCttctggaAAA (+7bp)

cmlc1             TTCCAGAGACACCCAAAGAGCCCGAAGTGGACCTGAAAAGCGTCCCGCTG
cmlc1(-16bp)      TTCCAGAc-----------------AAGTGGACCTGAAAAGCGTCCCGCT (-17, +1bp)
cmlc1(+29bp)      AGACACCCAAAGAGCCCGaaaagctctagagactccagagacatccaAAG (+29bp)

tnnc1a            ATGAACGACATCTACAAAGCAGCGgtgagtc
tnnc1a(-5bp)      ATGAACGACATCTA-----CAGCGgtgagtc        (-5bp)
tnnc1a(+13bp)     ATGAACGACATCTACAtcacctacatccacat---CAG (-3, +16bp)
```

C

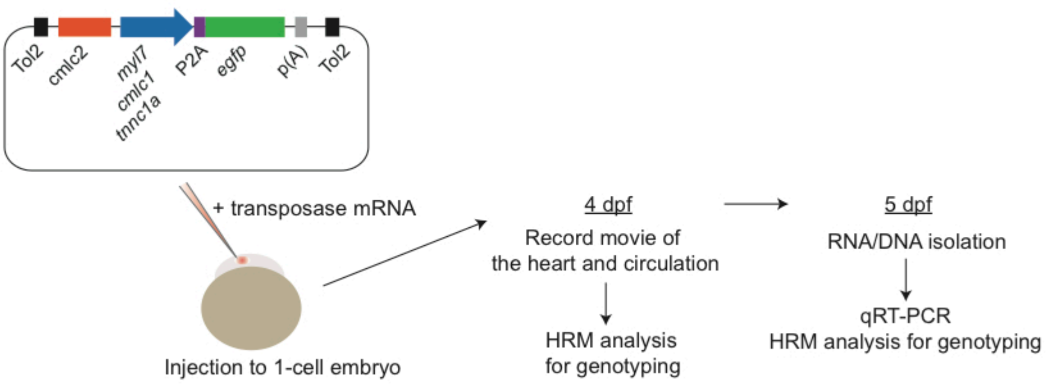

D

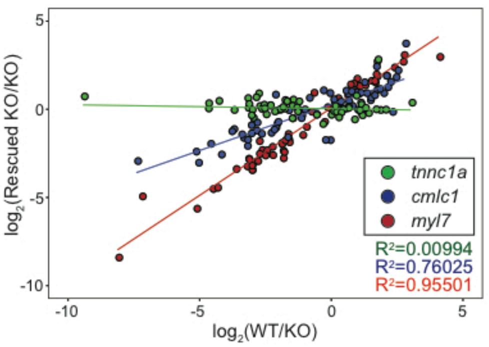

Biology Open • Supplementary information

Figure S4. Cardiomyocyte-specific gene mutants and their genetic rescue. A. CRSIPR/Cas9 mediated mutation strategies for myl7, cmlc1 and tnnc1a. **B.** Mutations introduced in myl7, cmlc1 and tnnc1a genome. **C.** Genetic rescue strategy. **D.** Correlational plots for WT/Mutant and Rescued/Mutant. Complete (R^2=0.95501), partial (R^2=0.76025) and no rescues (R^2=0.00994) by myl7 promoter-driven re-introduction of myl7 (n=8), cmlc1 (n=3 for the rescue experiment, n=4 for the control cmlc1$^{-/-}$), WT and tnnc1a (n=8), respectively.

Biology Open • Supplementary information

Figure S5

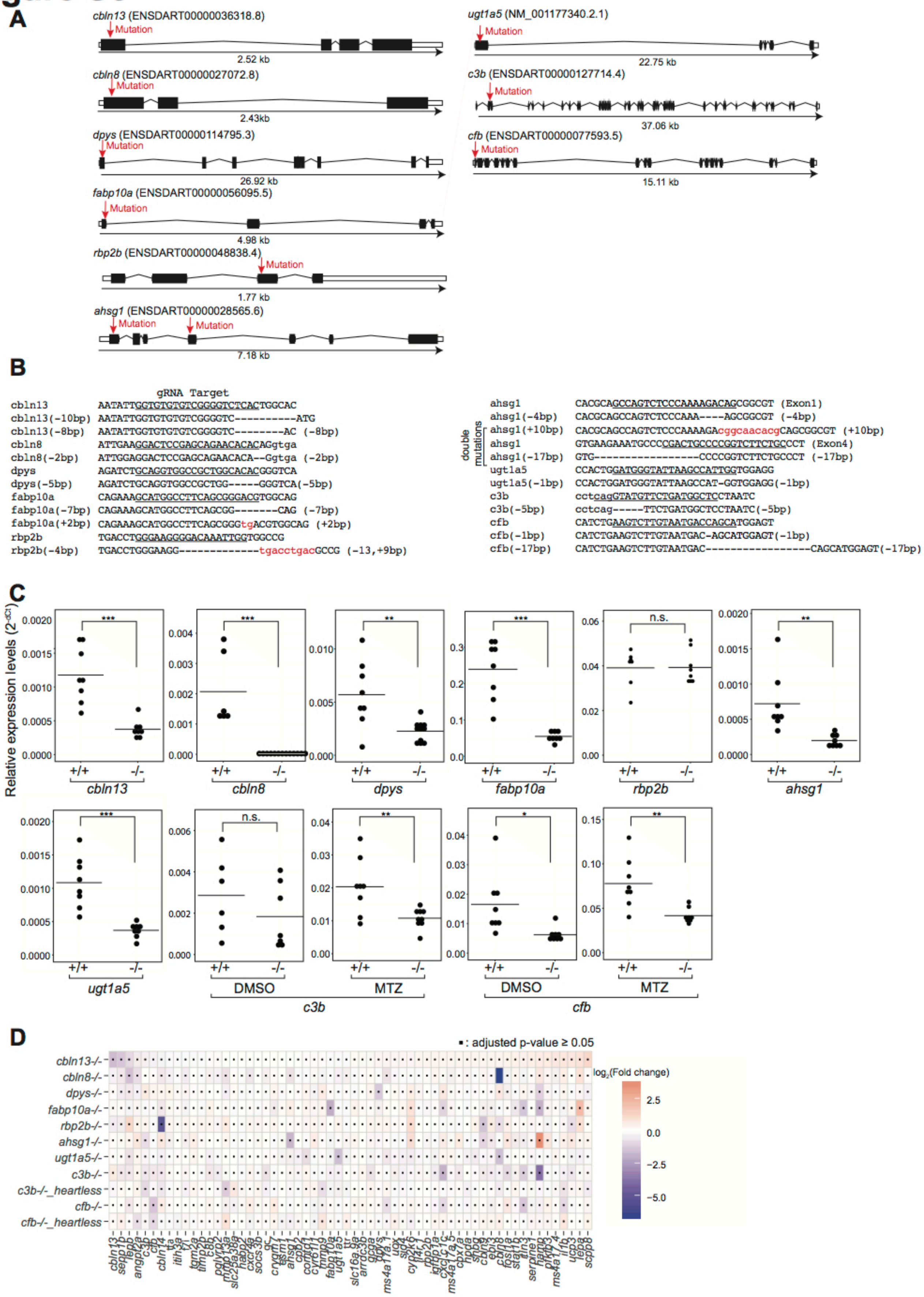

Biology Open • Supplementary information

Figure S5. Genetic study of a possible cross-regulations among liver genes. A. CRSIPR/Cas9 mediated mutation strategies for cbln13, cbln8, dpys, fabp10a, rbp2b, ahsg1, ugt1a5, c3b, cfb. **B.** Mutations introduced into their genomes. **C.** qRT-PCR analysis to validate NMD (non-sense mediated decay) for each gene mutation. *p<0.05, **p<0.01, ***p<0.001, n.s.: not significant, student-t test. n=8 (cbln13$^{+/+}$), n=8 (cbln13$^{-/-}$), n=6 (cbln8$^{+/+}$), n=13 (cbln8$^{-/-}$), n=8 (dpys$^{+/+}$), n=11 (dpys$^{-/-}$), n=8 (fabp10a$^{+/+}$), n=8 (fabp10a$^{-/-}$), n=7 (rbp2b$^{+/+}$), n=8 (rbp2b$^{-/-}$), n=8 (ahsg1$^{+/+}$), n=8 (ahsg1$^{-/-}$), n=8 (ugt1a5$^{+/+}$), n=8 (ugt1a5$^{-/-}$), n=6 (c3b$^{+/+}$_DMSO), n=7 (c3b$^{-/-}$_DMSO), n=8 (c3b$^{+/+}$_MTZ), n=9 (c3b$^{-/-}$_MTZ), n=8 (cfb$^{+/+}$_DMSO), n=8 (cfb$^{-/-}$_DMSO), n=8 (cfb$^{+/+}$_MTZ), n=8 (cfb$^{-/-}$_MTZ). **D.** Heatmap representations for the gene expression patterns in each mutant. For c3b and cfb mutants, the results for both DMSO- (mutant) and MTZ- (mutant and "heartless" double) treated larvae are shown. ■$p \geqq 0.05$, student t-test followed by Benjamini-Hochberg procedure to correct errors for the multiple tests. n=6 – 13.

Biology Open • Supplementary information

Figure S6

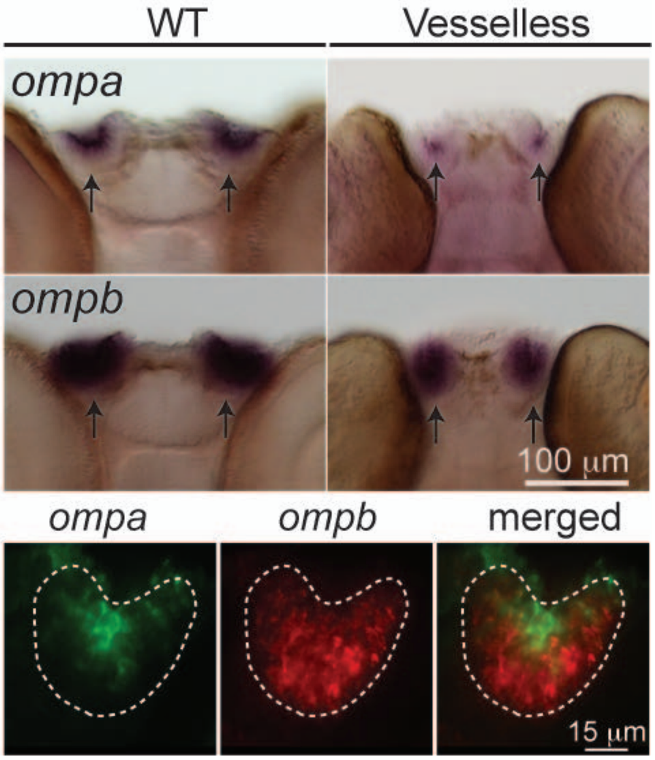

Figure S6. Ompa is expressed in a sub-domain of the olfactory bulb. WISH patterns of olfactory-specific genes, ompa and ompb. Scale bar, 100 µm. Double WISH staining for ompa and ompb (bottom). Green: ompa. Red: ompb. Scale bar, 15 µm. Dotted white line, olfactory epithelium.

Biology Open (2017): doi:10.1242/bio.030254: Supplementary information

Figure S7

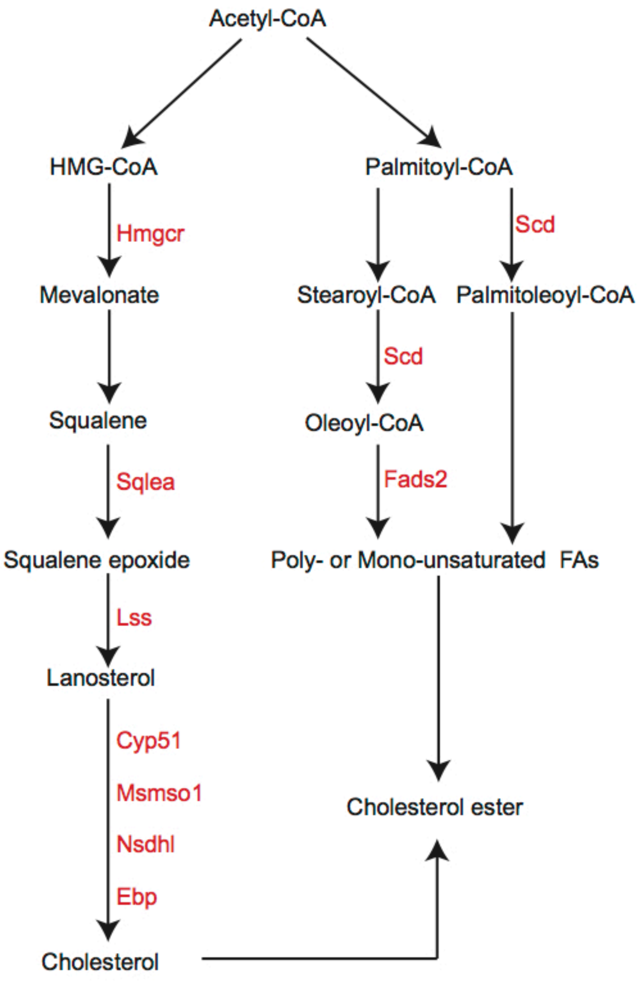

Figure S7. The pathways for cholesterol biosynthesis. The genes specifically increased in "vesselless" are indicated in red.

Biology Open • Supplementary information

Figure S8

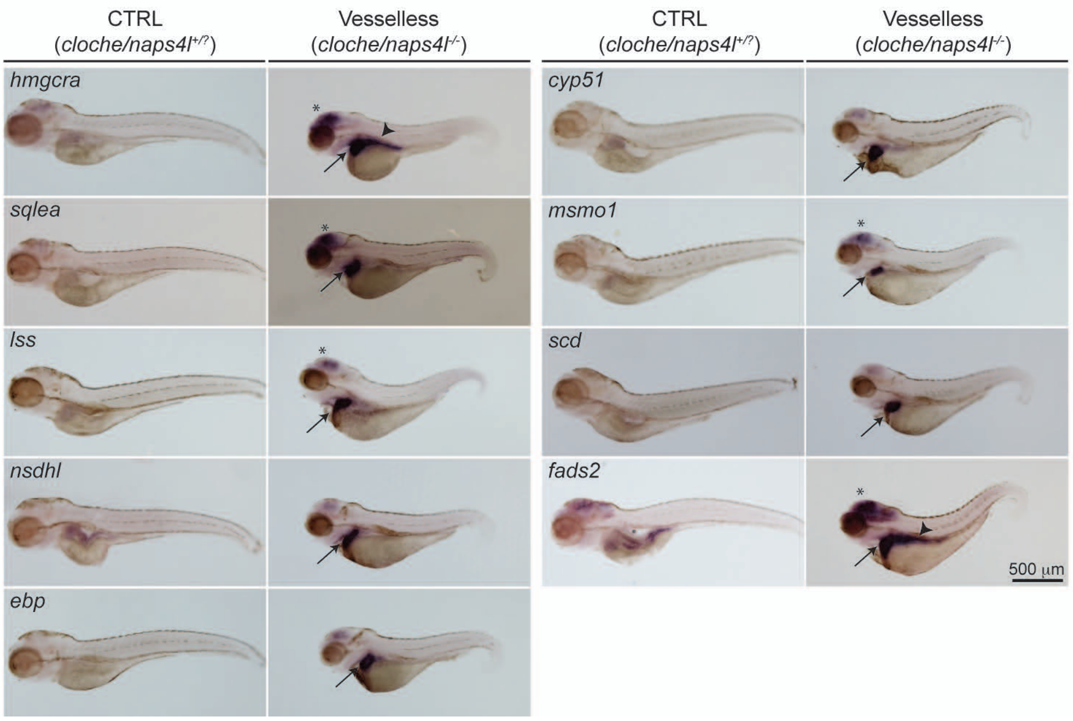

Figure S8. Expression patterns of genes encoding cholesterol biosynthesis. WISH patterns for each gene are shown. Arrows: Liver. Arrowheads: Intestine. *: Brain. Scale bar, 500 µm.

Biology Open • Supplementary information

Figure S9

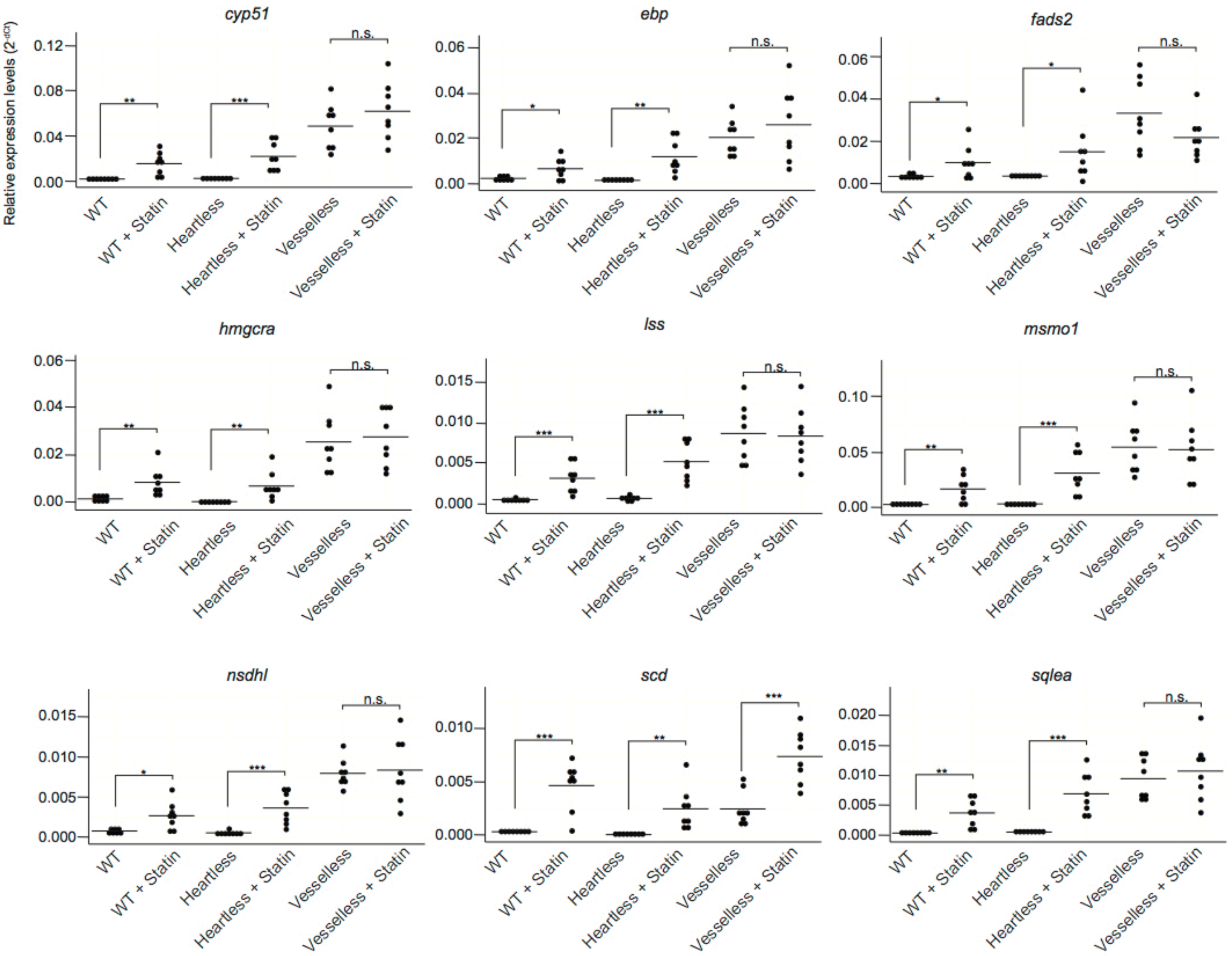

Figure S9. The expression of the genes encoding enzymes for cholesterol biosynthesis in statin-treated larvae. The qRT-PCR results showing the expression of each gene in control (WT), atorvastatin-treated control (WT+Statin), ”heartless” (Heartless), atorvastatin-treated “heartless” (Heartless+Statin), “vesselless” (Vesselless) and atorvastatin-treated “vesselless” (Vesselless+Statin) larvae . *p<0.05, **<0.01, ***p<0.001, n.s., not significant, n=8.

Biology Open • Supplementary information

Figure S10

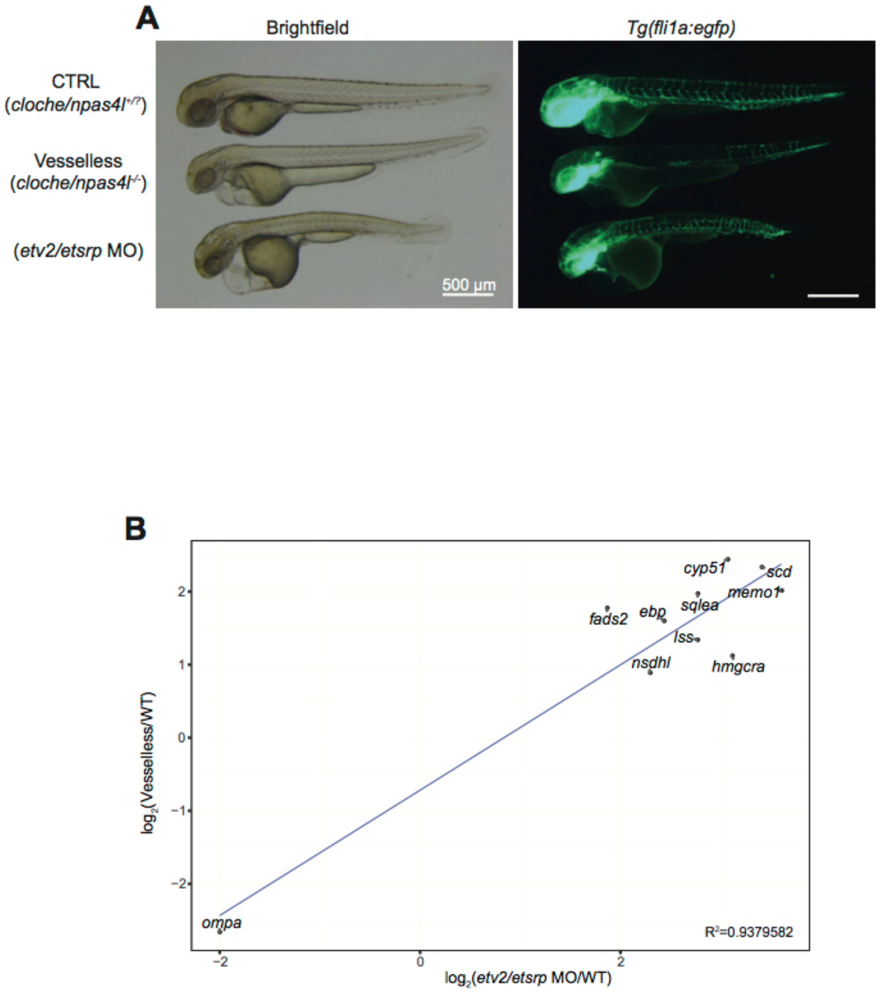

Figure S10. Characterization of etv2/etsrp morphant. A. Reduced vasculature in etv2/etsrp morphant. The vasculature of *cloche/npas4l*$^{+/?}$, *cloche/npas4l*$^{-/-}$ (i.e., “vesselless”) and etv2/etsrp morphant is compared by fli1a:egfp signals. Scale bars, 500 µm. **B.** Correlational plot of “vesselless” and etv2/etsrp morphant (etv2/etsrp MO). R^2=0.9379582. n=19 (WT), n=26 (MO), n=8 (“vesselless”), n=8 (sibling control).

Biology Open • Supplementary information

Figure S11

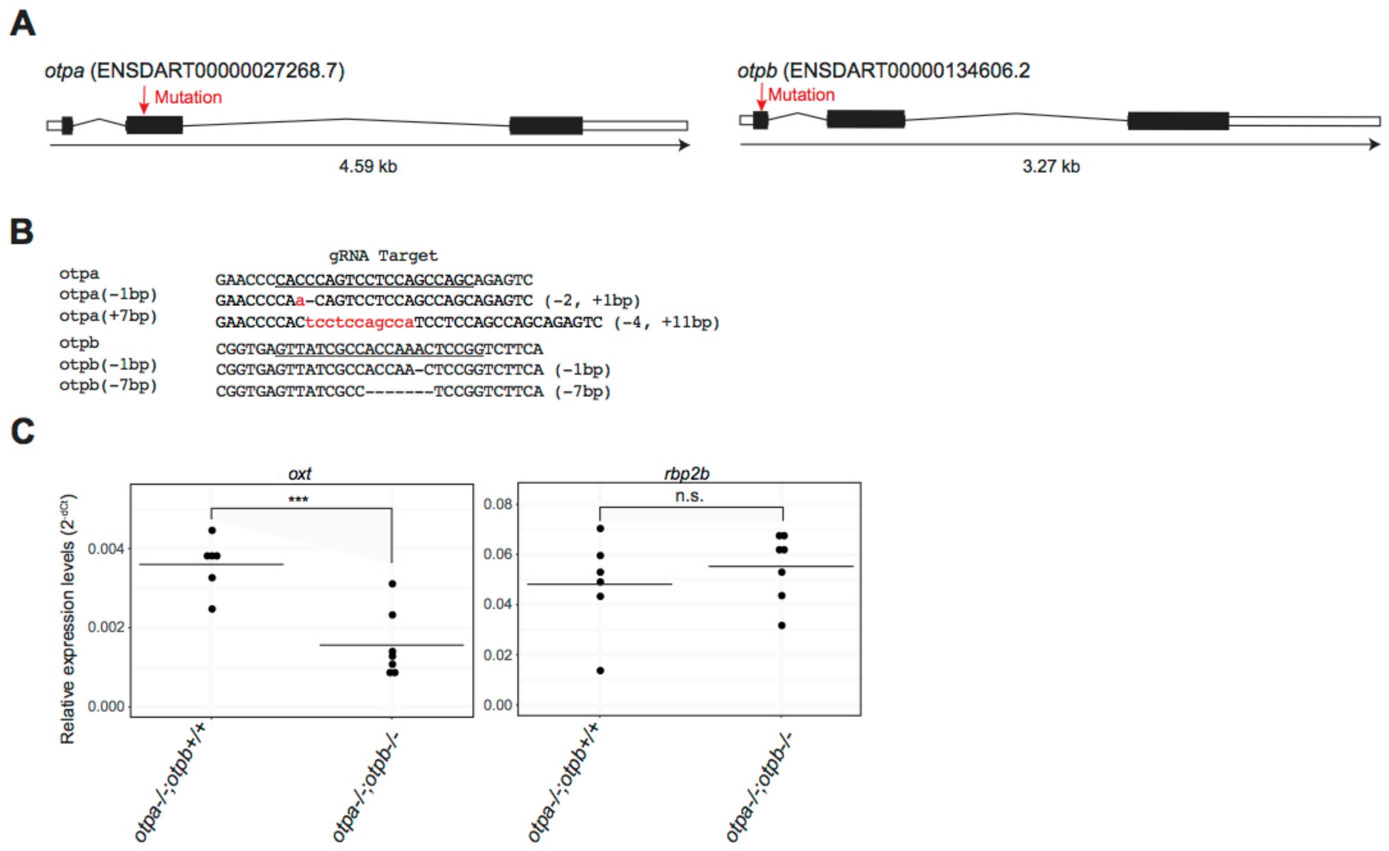

Figure S11. Characterization of double mutant for otpa and otpb genes. A. CRSIPR/Cas9 mediated mutation strategies for otpa and otpb genes. **B.** Mutations introduced into otpa and otpb genome. **C.** qRT-PCR analyses of oxt and rbp2b expressions. ***p<0.001, n.s., not significant. Student-t test. n=6(WT), n=7(otpa$^{-/-}$;otpb$^{-/-}$).

Biology Open • Supplementary information

Table S1. This excel table contains qRT-PCR data (Fig. 1C, Figs. 2B, 2C, Figs. 3C, 3F, Fig. 4C, Fig. 5B, Fig. 6B, Figs. S1D, S4D, S5C, D, S9,S10B, S11C) used in this study.

Click here to Download Table S1

Table S2. This excel table contains a list of genes, primers, riboprobes, gRNAs and MO sequences used in this study.

Click here to Download Table S2

Biology Open • Supplementary information

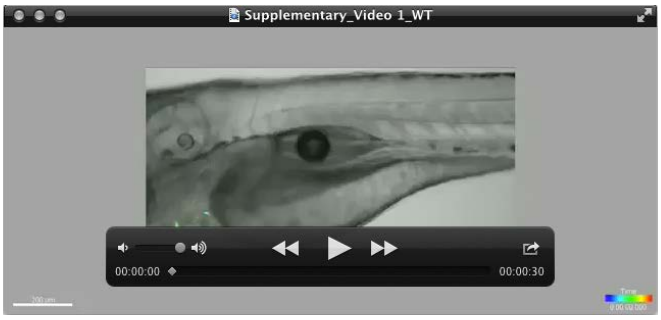

Movie S1. This video shows cardiac contraction and circulation of wild type.

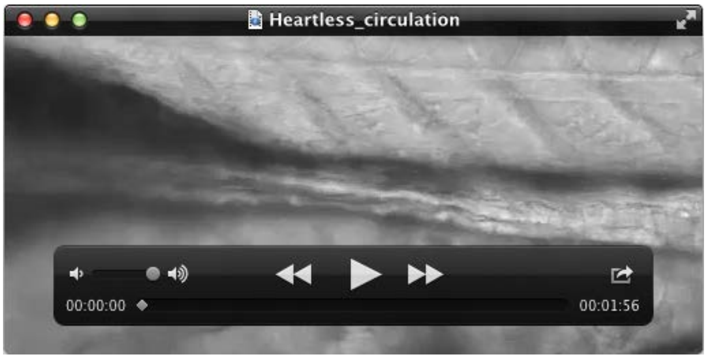

Movie S2. This video shows the lack of circulation of “heartless”.

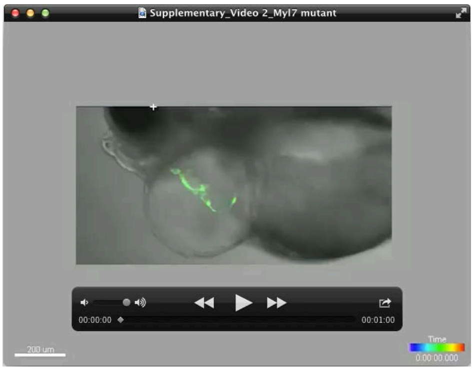

Movie S3. This video shows the lack of cardiac contraction and circulation of myl7 mutant.

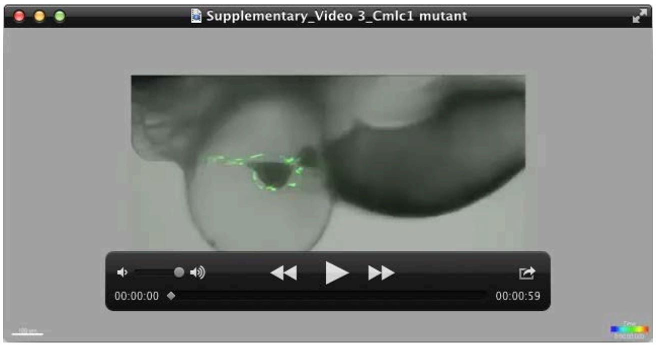

Movie S4. This video shows “shuddering” cardiac movement and the lack of the circulation of cmlc1 mutant.

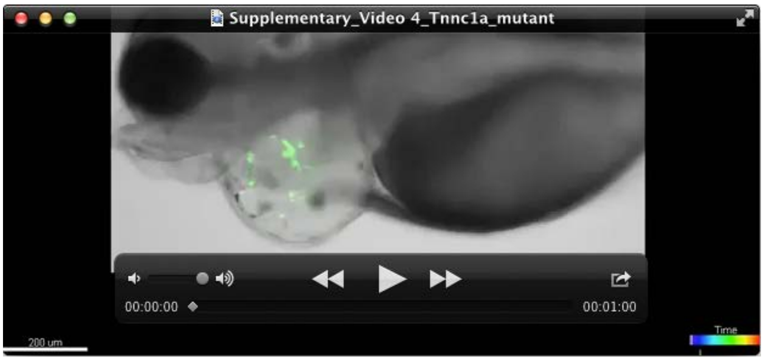

Movie S5. This video shows aberrant cardiac contraction and circulation of tnnc1a mutant.

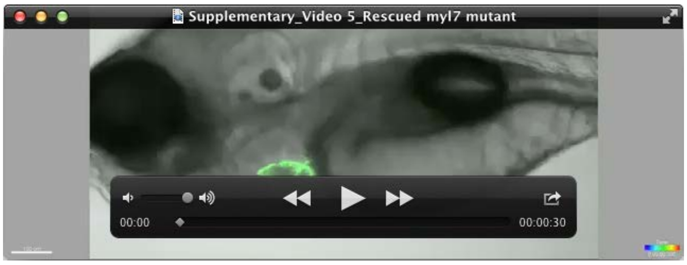

Movie S6. This video shows normal rescued cardiac contraction and circulation of the myl7 mutant.

Biology Open • Supplementary information

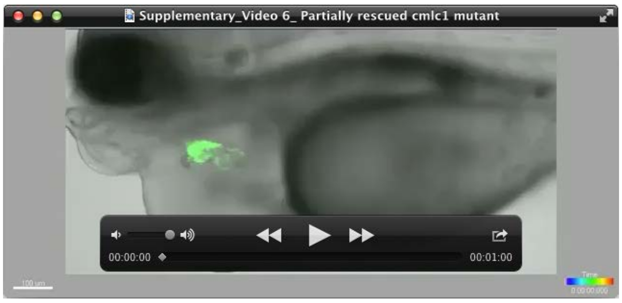

Movie S7. This video shows partially rescued cardiac contraction but no circulation of the cmlc1 mutant.

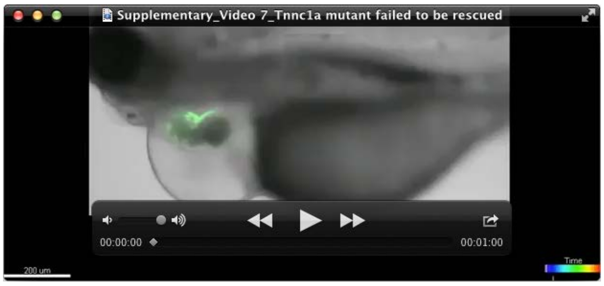

Movie S8. This video shows the lack of cardiac contraction and circulation of the tnnc1a mutant following the re-introduction of tnnc1a via the myl7 promoter.

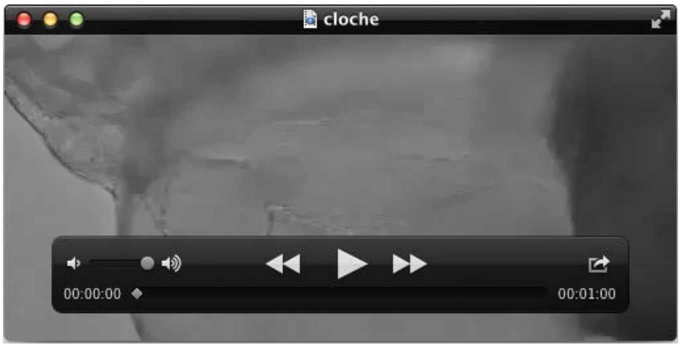

Movie S9. This video shows cardiac contraction without endocardium but no circulation of "vesselless" (*cloche/npas4l$^{-/-}$*).

Biology Open • Supplementary information

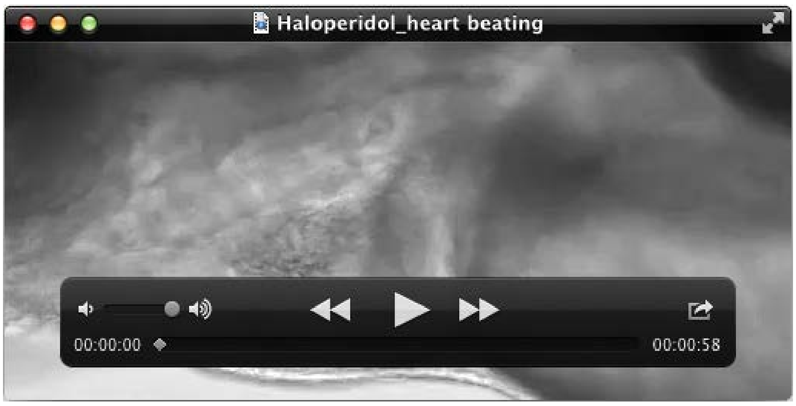

Movie S10. This video shows the lack of cardiac contraction and circulation of the haloperidol-treated (5 hrs) larvae (4.5 dpf).

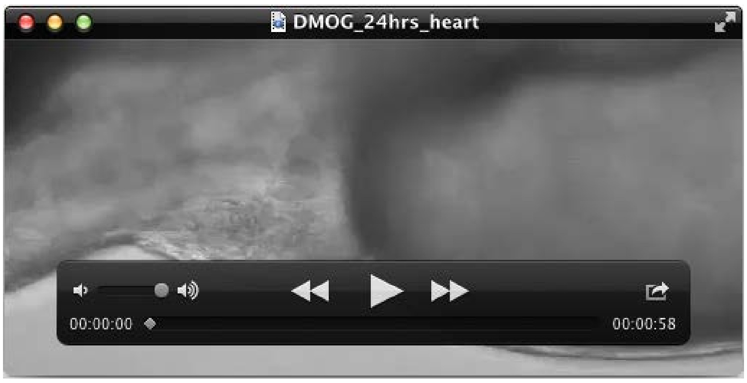

Movie S11. This video shows the DMOG-treated (24 hrs) larvae (4.5 dpf)

Biology Open • Supplementary information
